# The lived experience of gender dysphoria in autistic young people: a phenomenological study with young people and their parents

**DOI:** 10.1007/s00787-022-01979-8

**Published:** 2022-04-04

**Authors:** Kate Cooper, Catherine Butler, Ailsa Russell, William Mandy

**Affiliations:** 1 Centre for Applied Autism Research, Department of Psychology, University of Bath, Bath, BA2 7AY UK; 2 Department of Psychology, University of Exeter, EX4 4QG Exeter, UK; 3 Department of Clinical, Educational, and Health Psychology, UCL Research, Gower Street, London, WC1E 6BT UK

**Keywords:** Autism, Gender diversity, Gender dysphoria, Adolescents, Transgender

## Abstract

G*ender dysphoria* is distress in relation to incongruence between an individual’s gender and sex assigned at birth. Gender clinics offer support for gender dysphoria, and there is a higher prevalence of autism in young people attending such clinics than in the general population. We aimed to investigate the lived experiences of autistic young people who have experienced gender dysphoria, and their parents, using a multi-perspectival IPA design. Young autistic people aged 13–17 years (*n* = 15), and their parents (*n* = 16), completed in-depth interviews about the young person’s experience of gender dysphoria. We analysed each individual transcript to generate individual themes, and for each of the dyads, developed themes which acknowledged the similarities and differences in parent–child perspectives. The first superordinate theme was *coping with distress* which had two subordinate themes; *understanding difficult feelings and focus on alleviating distress with external support*. This theme described how young people were overwhelmed by negative feelings which they came to understand as being about gender incongruence and looked to alleviate these feelings through a gender transition. The second superordinate theme was *working out who I am* which had two subordinate themes: *the centrality of different identities and needs* and *thinking about gender.* This theme described how young people and their parents focused on different needs; while young people more often focused on their gender-related needs, parents focused on autism-related needs. We conclude that young people and parents may have different perspectives and priorities when it comes to meeting the needs of autistic young people who experience gender dysphoria.

## Introduction

Transgender and gender diverse young people have gender identities which are incongruent with their sex assigned at birth, and some young people experience distress in relation to this incongruence, or gender dysphoria. Gender Dysphoria in adolescents is defined in the Diagnostic and Statistical Manual of Mental Disorders (5th ed.; DSM-5; [3] as a significant incongruence between an individual's gender identity and assigned sex with associated distress or impairment. In this paper, we use the term *gender dysphoria* to refer to any distress associated with gender incongruence, rather than to the DSM-5 diagnosis.

Young people experiencing distress associated with gender incongruence can access specialist healthcare at gender clinics, which offer comprehensive assessment of Gender Dysphoria, and possible referrals for gender-affirming hormonal treatments [42]. It is important to note that not all young people attending gender clinics will meet diagnostic criteria for Gender Dysphoria, nor will they all want to undertake a physical transition. Multiple gender journeys are to be expected; some young people may not identify as transgender or gender diverse by adulthood; some will undertake a social transition only (e.g. change their gender presentation, names and pronouns); and some will have an endocrinology assessment with a view to receiving gender-affirming treatments [30].

In the UK, transgender healthcare is provided for children and young people in a National Health Service (NHS) clinic, called the Gender Identity Development Service (GIDS). There are three stages of intervention [7]: first, a psychosocial therapeutic assessment over a minimum of 6 months, during which a Gender Dysphoria assessment is undertaken. The next stage is open to those who receive a diagnosis of Gender Dysphoria and involves a referral to the paediatric endocrinology team to consider the use of hormone blockers to stop the progression of puberty. The third stage is open to those whose Gender Dysphoria persists and involves the use of cross-sex hormones to change the young person’s body in line with their gender identity. Approximately 38–40% of young people referred to the UK gender service go on to be referred to see an endocrinologist [7]. For young people under the age of 16, parental consent is required to progress to physical interventions, alongside clinician assessment of the young person’s capacity to consent. Young people aged over 16 years are deemed to be able to consent to their own treatment. There is evidence that hormonal treatments are effective in achieving their intended physical effects, but there is not enough evidence to make conclusions about their psychological impact, and the quality of this evidence is generally poor [8]. Research indicates that parental support is important to the psychological well-being of gender diverse young people [28], with evidence suggesting that young people with more accepting parents have better mental health [41].

Rates of autism are higher in young people accessing gender clinics than in the general population. The global prevalence of autism is thought to be 1% [4], and a study of English schoolchildren found a prevalence of 1.76% [27]. In the UK gender service, 15% of young people who attended an assessment in 2015 were autistic [25], compared to 8% in the Netherlands clinic [11], and 26% in the Finland clinic [18]. Moreover, a large-scale study in adults found that transgender and gender diverse adults were 3–6 times more likely to be autistic than cisgender people [40]. It is possible that other neurodevelopmental conditions are over-represented in gender diverse young people, with one study finding higher rates of gender variance in participants who were autistic and those who had ADHD compared to those with medical neurodevelopmental conditions [31].

Research in this area has frequently investigated the co-occurrence of autism and gender dysphoria through the use of cross-sectional questionnaire designs, often drawing on single items from questionnaires such as the child behaviour checklist (e.g. [23]. There have been initial attempts to investigate factors explaining the relationship between autism and gender diversity, although the available research is cross-sectional and so it is not possible to assess causal mechanisms. Some researchers have investigated the role of social differences in gender dysphoria. For example, van der Miesen et al. [37] found that autism features were elevated in children and adolescents with gender dysphoria compared to a control group. This was across all domains, including in social interest and contact, and difficulties in understanding social information. This suggests that the social differences found in autism might be associated with gender dysphoria. Other research in adults has investigated the relationship between sensory differences and gender dysphoria, with one study finding that a sample of mostly non-binary autistic adults had lower scores in visual and auditory hypersensitivity as compared to cisgender autistic adults [38]. Another study found that transgender adults had higher levels of sensory sensitivity than cisgender adults [40]. Research into executive functioning differences has found that children and adolescents with gender dysphoria have higher executive dysfunction scores when compared to controls [2]. Furthermore, in a sample of transgender young people, those on the autism spectrum had higher executive dysfunction compared to non-autistic young people [35]. Finally, other researchers have suggested that biological differences [13] may also be relevant to the development of gender identity in this population.

Given the high number of autistic young people attending gender clinics, it is crucial to better understand the lived experience of this group. However, there is little research focused on the lived experiences of gender diverse and autistic young people except for two well-characterised studies from the USA. One focused on developmental trajectories of gender dysphoria in autistic young people [32], and another on the development of a clinical programme to support gender diverse and autistic young people [33]. These studies, which used participatory methods and framework analysis, indicated that autistic young people had vivid experiences of gender dysphoria which they hoped would be resolved through gender-affirming treatments, that they identified autism as impacting their ability to understand and communicate about gender dysphoria; that their awareness of gender diversity developed over time; that they worried about being ostracised due to their gender identities, or that their gender would be dismissed due to their autism; and imagined a more positive future for themselves following gender-affirming treatments [32]. Young people and parents indicated a preference for a clinical support group aimed at meeting gender—as well as autism—related needs and fostering social connections with other gender diverse autistic young people, alongside support for parents [33]. Aside from these studies, there is a paucity of research into the lived experience of gender dysphoria in autistic young people, and none from outside the USA. Moreover, no studies have investigated the lived experience of gender dysphoria from the perspective of both young people and their parents. This is essential given the role that parents play in supporting the mental health of gender diverse young people.

In this study, we aim to answer the questions: how do autistic young people experience gender dysphoria? How do young people and parents perceive the intersection of autism and gender dysphoria?

## Method

### Methodological approach

Interpretative Phenomenological Analysis (IPA) uses qualitative techniques to understand the lived experience of a specific phenomenon, in this case, gender dysphoria in autistic young people. IPA is an idiographic approach, focusing on the experiences of individual participants, and aims to understand the meaning of these experiences [29]. Within IPA, the researcher is actively making sense of the participant’s descriptions of their experiences and must maintain an awareness of their own influence on the analysis. IPA has been found to be a useful tool when working with autistic people and helps to increase the focus on autistic rather than non-autistic researcher perspectives [15, 22]. IPA is an interpretative approach and therefore findings are limited to a detailed understanding of the particular experiences of a specific group, with no aim to explain or suggest causal mechanisms regarding the phenomena studied [36]. Therefore, as a research team, we aimed to maintain a reflexive stance, including an awareness of the limitations of our chosen method. The research team for the current study were all non-autistic, cisgender researchers and clinicians, and this positionality was considered throughout the analytic process.

### Participants

We aimed to recruit young people and parent pairs into the study. In total, there were 14 young person-parent pairs, 1 individual young person, and 2 individual parents (*n* = 31). Therefore, 17 young people were the focus of the study. Young people were aged 13–17 years and had a clinical diagnosis of Autism Spectrum Disorder which was verified by viewing diagnostic reports written by a qualified health professional. Young people identified as transgender, had experienced distress in relation to their gender incongruence, and had asked for support from a professional regarding this distress. This broader definition of gender dysphoria was selected in recognition of the potentially different developmental trajectories of gender identity in adolescence, and the shifting conceptualisations of gender dysphoria over time [5]. This strategy was endorsed by the Patient and Public Involvement group for the study and allowed for the recruitment of young people who self-identified as experiencing gender dysphoria, and who were sufficiently distressed to seek support from a professional about this.

We used purposive sampling so that participants were varied in terms of geographical location within the U.K., and age and stage of transition, leading to a large sample size for an IPA study. Participants were recruited from U.K. National Health Service (NHS) gender and mental health services and from community groups for LGBT + young people and their families. See Table 1 for a summary of characteristics of young people in the study, and Table 2 for parents included in the study.

**Table 1:** Young person demographic characteristics (*n* = 17)

Gender	*n*	%
Male	10	59
Female	3	18
Non-binary/genderqueer	4	24
Sex assigned at birth		
Male	3	18
Female	14	82
Sexuality		
Straight	3	18
Lesbian or gay	3	18
Bisexual	6	35
Questioning	2	12
Other	3	18
Ethnicity		
White British	15	88
Mixed	2	12
Stage of gender journey		
Discussed with a professional	2	12
On gender service waitlist	6	35
Ongoing assessment at gender service	3	18
Hormones prescribed privately	3	18
Hormones prescribed on NHS	3	18
Recruited from		
Gender service	7	41
Mental health service	6	35
Community	4	24

	Mean	Range
Age in years	15	13–17
Age realised trans	10	4–16
Age when sought help from a professional for gender dysphoria	13	6–16

**Table 2:** Parent demographic characteristics (*n* = 16)

Gender	*n*	%
Male	2	13
Female	14	88
Sexuality		
Straight	16	100
Ethnicity		
White	15	94
Mixed	1	6

	Mean	Range
Age in years	48	42–55

### Procedure

We received ethical approval for the study from the Health Research Authority (19/NE/0265). For participants recruited in the NHS, they were introduced to the study by their routine clinician, who assessed their eligibility to take part before gaining consent to pass on their contact details to the research team. Participants from community settings directly contacted the research team after seeing the study advert. We then assessed their eligibility to participate (see above). We invited participants to meet with a researcher in-person, on the phone, or on an online video call, to give fully informed consent, or assent, for young people under the age of 16. Parents provided consent for their children under the age of 16 to participate. After consenting, participants completed a demographic questionnaire; parents provided brief demographic information about themselves, and full demographic information about their children, if their child had not already provided this data (see Table 1).

Next, young people and parents agreed who would be interviewed first, and whether the young person wished to be interviewed alone or with their parent present. Young people were generally interviewed alone, unless they felt more comfortable with their parent present. This was an important autism adaptation to the procedure, although may have limited the amount of information shared by young people when their parent was present. Parents were generally interviewed alone, although as interviews took place online and during school closures due to the COVID-19 pandemic, sometimes the young person participant or siblings entered the room during the interview. The young person and parent interviews were then conducted in turn; both interviews focused on the young person’s experience of distress linked to gender incongruence, and how they had sought help for this. The interviews were conducted between January and October 2020 and lasted between 25 and 85 min. The topic guide covered the young person and parent’s first memories of thinking about the young person’s gender identity; the experience of being autistic and experiencing distress about gender incongruence; relationship between mental health and gender; seeking help for gender dysphoria with a focus on healthcare settings. As recommended for IPA studies, open questions were used in the first instance [29], although closed prompt questions were used to help participants who were struggling to articulate their experiences.

Many participants required adaptations to the interview, including having their parent present during the interview, responding to the questions by text message, not having their video on during video calls and having a fiddle toy to play with. The patient and public involvement group who provided advice on the conduct of this study were clear that some autistic participants would feel more comfortable, and would be able to express themselves better, through remote contact without a video, and indeed some participants did request this. However, phone interviews have been criticised within qualitative research as potentially leading to a more limited and less-rich dataset [26]. Some interviews with young people were rather brief and did not provide as rich a dataset as some other interviews, but all the interviews proved useful in helping to answer the research question.

We audio recorded the interviews using a digital recorder, and recordings were professionally transcribed by an external company, and checked for accuracy by the first author. Young people were reimbursed with a £25 shopping voucher, and parents with a £15 voucher, and sent a debrief sheet following participation.

### Analysis

We used a multi-perspectival IPA design in this study, with parents and young people both being interviewed about their perspectives on gender dysphoria in autistic young people [20]. At the level of each individual transcript, analysis involved capturing descriptive, linguistic and conceptual elements of the transcript. It was notable that sometimes participants used idiosyncratic language that was difficult to interpret, in line with social communication differences in autism. There were, therefore, fewer ‘linguistic’ comments than there might be in an IPA analysis with non-autistic participants. Using the initial notes, we developed themes which had related ideas, those with oppositional ideas, themes with ideas that had a similar function within the transcript, and themes which centred on contextual factors which were meaningful to participants. Once each transcript had been analysed in this way, we developed themes for each parent and young person pair (*n* = 14). Within each pair, themes were brought together by identifying consensus and conflict, finding reciprocal themes, identifying paths of meaning (shared or divergent meanings attributed to the same or different experiences) and finally identifying lines of argument within the analysis or "storying" the important themes within the analysis [20]. Finally, a similar process was used to identify overarching themes for the whole group of participants, considering the parent and young person pairs, along with the themes for the two individual parents and one individual young person who we interviewed. As there was a large sample-size for an IPA study, the number of participants who contributed to each theme was considered, and any theme that applied to more than half of the young people (*n* ≥ 9) was categorised as recurrent and described in the final analysis [29]. We used quotes from participants when they were an apt and succinct summary of the meaning of each theme, while also trying to ensure that the whole range of participants was represented through quotes. We prioritised using quotes from young people but when a parent articulated a theme more clearly than their child, and when parents tended to have different viewpoints to their children, we prioritised parent quotes. Participant numbers are provided, and young person participant numbers start with 1, and parent participant numbers start with 2.

We aimed to ensure that the analysis was credible, and to do this as a research team we discussed our positionality and assumptions before and during the project. The first author kept a reflexive diary during the research process and attended an IPA research group with other researchers. The analysis was critically evaluated in supervision and in the IPA group, thereby ensuring that the analysis was grounded in the transcripts and participants’ reported experience.

### Patient and public involvement group

A group of transgender autistic people and family members were consulted and formed a patient and public involvement group for the research project. These individuals were invited to contribute to the research by giving their opinions on the suggested research question and methods. They reviewed the study materials to ensure that they were adapted for young autistic people. The themes from the study were checked to ensure that they appeared credible and that they made sense to community group members.

## Results

### Coping with distress

This superordinate theme outlines the process undertaken by young people and their parents to make sense of the negative emotional experiences they were having, by identifying the causes of the distress and expressing these feelings. Once distress was identified as being due to gender incongruence, many young people understood gender-affirming treatments to be the solution to their distress, and this became their focus. Young people and their parents described the need for gender and mental healthcare to be adapted to meet their needs as autistic and gender diverse young people.

#### *Understanding difficult feelings (n* = *17)*

The first subordinate theme is about participants’ experiences of being overwhelmed by negative feelings which were hard to understand and express. Participants varied in the intensity of distress they described, and perceived causes of the distress, although almost all young people had negative feelings about the mismatch between their gender identity and bodies, and puberty was frequently cited as being very difficult. Participants also varied in their conceptualisation of distress i.e., the extent to which distress was due to gender incongruence, and how gender distress related to other types of distress.

Most participants spoke about significant difficult feelings young people experienced due to the mismatch between their gender identities and bodies. Participants described a range of emotional experiences; negative feelings about the body included “anger”, “anxiety”, and feeling “sad” (105). One young person felt trapped in his body, demonstrating how uncomfortable it was to inhabit his body and experience such significant distress about it: *“To put it short, it’s hell, it feels like I’m trapped, it’s something I wouldn’t wish on anyone. It’s not fun, it’s looking in the mirror and wanting to die, pretty much, because you hate yourself that much. I just want to be the opposite gender and it’s difficult” (104).* Many participants had negative experiences of puberty, with a noticeable increase in gender distress at this time, struggling as their bodies changed in unwanted ways, and the mismatch between body and gender identity increased: *“Puberty was really starting to take course, I really dipped which makes sense….it’s just my body was getting more and more masculine. My voice was getting deeper. I was getting taller. I was getting hairier and stuff and it just made me feel disgusted with myself” (105)*.

Parents most often became aware of their child’s gender dysphoria when their child had reached puberty. Parents were more likely to highlight distress linked to puberty than young people; one parent (204) highlighted the negative impact of early periods of their child, and another parent reflected on how sensory and gender needs combined to create distress around periods: *“because of him dealing with his monthly cycle….Yeah, he hated the smell of everything. He hated the mess. He’s always been a germophobe anyway. That has really messed with him.” (214)*. Other young people and parents pointed to the relationship between sensory overload due to autism and intense body distress, for example, 102 described the impact on sensory needs on gender dysphoria: *“It’s probably enhanced it a bit ‘cause I get sensory overload quite easily. When all my sensory stuff is heightened I notice my body a lot more and I’m not able to take myself away from it and rationalise it”.* Another non-binary young person’s negative feelings about their chest started part way through puberty, which made their parent question whether their gender dysphoria would continue in the long term: “*he does want it [chest] to look more flat but going back a year ago, she was quite proud of those little boobs growing … so you can see where I’m getting really confused.”(209).*

Not all participants experienced significant distress in relation to their bodies, with some participants describing mild or very specific dysphoria (e.g. just about the genitals). One non-binary participant described mild discomfort that they could not have a non-binary body, alongside a process of acceptance: “*And like with my physical appearance with my body and stuff I don’t mind having a female body but to me it’s not a female body if that makes sense because it’s just mine and I’m not female … realistically for me if I wanted a body to represent my gender then that’s basically impossible so sometimes I do feel uncomfortable with it” (108).*

Participants also described negative feelings due to factors other than gender incongruence and described a process of trying to make sense of what was causing the distress. For example, one young person discussed coming to understand the causes of his distress: “*it was gender but I didn’t quite know that yet and it was a lot of the bullying and I was just depressed in general*” (114). Many participants felt that dysphoria compounded other mental health issues: *“You isolate yourself and you’re panicking all the time so it’s a lot of anxiety and feeling alone, so it’s definitely not helped [my mental health]. I have problems on top of that, so I have really bad anxiety and other problems but it doesn’t help at all, it’s just like another weight on top of that which makes it a lot worse and aggravates it.” (109).* Parents also described extreme distress which came to be understood after efforts of the young person, parent and clinicians: “*most of [his] childhood – meltdowns have been really frequent. And certainly, at that time they were more frequent than they had been and way more aggressive. More violent. Violent to himself as well. Erm. And it was with my thinking and reading and the help of [gender clinician] and everyone at the clinic, helping to understand where that level of anxiety was coming from.”* (216). Not only were feelings difficult to understand, but they were difficult to express. Feelings were, therefore, sometimes expressed behaviourally, with one parent describing a “tornado” (217) of emotions with angry outbursts and after some serious incidents of self-harm. Young people also highlighted issues with expressing their distress: *“The main thing that has been the hardest to be autistic and have gender dysphoria is not really being able to express the bad feelings very much…I just couldn’t understand how to word it properly, if that makes sense? I would definitely be more on the physically emotional side than verbally saying what’s wrong” (102).*

#### *Focus on alleviating distress with external support (n* = *16)*

This subordinate theme describes how participants wanted to alleviate their experiences of distress through undergoing medical procedures to change their bodies in line with their gender identity, with some exceptions. Young people understood that making a physical transition relied on external support, which required parental and professional support, with this sometimes leading to feelings of frustration.

Most young people were highly motivated to access physical interventions, with puberty blockers, hormonal interventions and surgery frequently cited as the solution to their distress. Many young people expressed frustration when there was not a clear and timely plan for gender-affirming interventions, as these would provide relief from distress; *“…feels like I’m covered in like a thick coat of black paint that I can't get out of and when I’m sad like that, I feel trapped that I can't get out of it and there’s nothing I can do. With depression I can rewire my brain, I can't rewire my brain into not being trans, I have to wait for surgery which takes too long.” (104).* This quote shows that this feeling of being trapped was increased by the need for external support to access medical interventions. Two young people were not sure that they wanted a physical transition, such as a non-binary participant: “*I think medically and like physically there is nothing I can do about my gender because I can’t make my body more non-binary*” (108) and a male participant felt that his social transition had alleviated his dysphoria: “*I feel like I don’t need help. Like I said earlier, I don’t get very much dysphoria, so it isn’t much of an issue for me*” (112).

While most young people wished to make a physical transition, parents and young people did not always align in their viewpoints. Parents wanted to help reduce the distress experienced by their child, while also emphasising their desire to slow down the process and for their child’s gender identity to be settled before undergoing any irreversible treatments. This tension was described by one parent who felt torn between their desire to alleviate their child’s distress, and to help keep their gender options open: “*As painful as that is, I think [child]’s got to try and work out whether he can be happy without medical intervention, and I don’t mean ever, I can see that you don’t put a suicidal teenager on hormone blockers and hope that that’s going to make them happier” (204).* Some parents saw themselves as completely aligned with their child’s desire to make a physical transition, positioning themselves as advocates for their children to access gender-affirming treatments: *“I suppose when you’ve got an autistic child you’ve become an advocate and that becomes your life because you’re the advocate for your child and I think we became the advocate for getting the support for [child] for going forward with his gender as well”* (203).

Young people wanted clinicians and parents alike to acknowledge and help alleviate their distress; for most participants, this meant affirming their gender identity and helping facilitate a physical gender transition. Some young people and parents felt let down by services and described barriers to accessing this treatment. Many participants expressed frustration at long waiting times for gender consultations: “*Because [child] is 17 in a few weeks, it changes everything because then you won’t get seen as a juvenile, you won’t get seen in time then you’ll have to go through it all again. So I know that [child] is gonna be panicking ‘cause I am. I’m actually fuming. I feel really… um it’s been sort of pushed aside a lot I feel.” (210).*

Once they did reach gender services, participants described difficulties communicating and connecting with gender clinicians due to a clash of communication styles. One participant described going to great lengths to understand their clinician’s communication style but did not think this effort was reciprocated: “*But sometimes—I’m not very good at the airy fairy sort of emotional talk they want to do. So they were not particularly receptive to me at first… they didn’t really – I don’t think they liked me very much.*” (107) There were also practical barriers including busy waiting areas, changing clinicians, travel to clinics, and sensory overload in both gender and mental health clinic settings, for example one participant anticipating their first appointment at a gender clinic said: “*it’s just getting there, that’s the main issue for me*.” (115).

### Working out who I am

This superordinate theme describes participants’ ways of making sense of their differences, with young people more often prioritising gender identities and related needs, and parents more often focused on autism identities and needs. Autism was conceptualised as impacting on thinking about gender by some parents, while some young people discussed the interaction between autism and the ways they thought about gender.

#### *The centrality of different identities and needs (n* = *17)*

This subordinate theme referred to participants’ different viewpoints on their identities and current priorities and needs. Most young people described feeling socially different to others, and participants had different perspectives on this. While parents more often focused on their child’s social differences and needs linked to autism, young people’s narratives generally centred their gender identities, while autism was often unimportant to their sense of self.

Young people described feeling different to other people their own age. Participants described being bullied, feeling isolated and like an outsider linked to social and gender differences, for example, 101 said *“when I got to secondary school I stood out quite a lot compared to other people. I was kind of singled out quite a lot so I had to try and fit in kind of, gender wise…I was kind of quiet and isolated and stuff so I would get picked on for like being quiet …making friends was really hard so that was for me tough as well*”. Parents and young people alike spent time trying to make sense of the young person’s differences and needs. In doing this, parents were more likely to centre needs linked to autism; *“I’ve always had an inkling that there was some kind of autism there. Getting it diagnosed is taking a long time but we’ve done it, but I think it was connected a lot of to the anxiety and to feeling that he wasn’t what he should… what in his mind he should be and I think… and it became a release the self-harming became a release to try and just release some of that… that self-doubt*” (201). Parents highlighted the need for a comprehensive understanding of their child’s needs, which included appropriate diagnoses to help facilitate understanding. Some parents described how they felt their children were making sense of themselves and social differences through the lens of gender diversity, while they thought autism may be more relevant: *“Going back to the identity thing is she trying out different identities when she feels more insecure about how she’s coming across socially? Does she try out different identities and is gender identity just part of that – I don’t know – without wanting to sound dismissive, I think that’s something that I perhaps need to consider.” (213).*

In contrast, many young people did not feel a strong connection with their autism label, for example one young person said “*Well, I was diagnosed [with autism] and I just dealt with it. I didn’t really care about it that much.” (117).* There were exceptions, for example one participant briefly mentioned feeling *“part of the [autism] club” (104).* However, many young people focused on their gender identities and needs within the interview as their focus and central identity; “*I’m still struggling with accepting myself as being trans. That’s one of my main issues at the moment*” (111). Some young people were keen to keep a separation between discussion of their autism and gender identities, with one young person making it clear that they felt this was necessary for their gender identity to be seen as valid *“I know that it’s a stereotypical thing that a lot of autistic people are trans….and I thought that now I also have that label people might not see me as valid as they would do if I didn’t have the autism… it felt like I wouldn’t be taken as seriously as it’s a typical autistic thing instead of an actual separate thing to autism.”*(102).

#### *Thinking about gender (n* = *17)*

Young people and parents conceptualised gender from a range of different perspectives. Young people most often conceptualised gender as a central, and real, part of the self, which they were certain about. Young people were less likely than parents to describe the impact of autism on their conceptualisation of gender, although spoke about how they interacted, and sometimes about the benefits of such interactions. Parents were more likely to describe how autism influenced their child’s thoughts about gender, often citing the influence of black and white thinking.

Young people sometimes described how thinking styles associated with autism interacted with their gender identity. One young person stated*: “I always thought it might be the autism affects me in the trans thing but it’s more that the trans thing has affected the autism if that makes sense. I used to think really black and white. Being trans has kind of made me stop doing that. I’ve had to move past it*” (105). Parents more often described the influence of black and white thinking linked to autism and noted the difficulties in the ‘grey’ areas of being transgender in terms of their children’s bodies not matching their identities. One parent described this in relation to a resistance to thinking about gender fluidity; *“Those conversations about fluidity have got shorter and shorter because it distresses [child]. That’s what I mean – it’s almost like he’s thinking, ‘You know. It’s black or white. It’s on or off. I identify here.”* (216). Other parents highlighted rigid thinking about what it meant to be a girl or a boy; “*I think the black and white … he likes playing with articulated traditionally boys toys, therefore, I must be a boy, I don’t like traditional girl things, therefore I must be a boy. And I think that thread runs through a lot with life really*.” (206). One parent linked black and white thinking to decisions around physical interventions; *“With autism, it’s black or white. ‘I’m doing this and I’m doing it now’. It’s like, no, you need to slow down a bit. You can’t always rationalise with him about it. Well, ‘I’ve decided, and I’m getting it sorted’.” (211).*

Some young people emphasised the importance of reaching 'certainty' about one's gender identity, asserting that their gender identity was an internally known and certain fact, and that this influenced decision-making; *“I’d waited quite a while so as soon as I came out it was the question if I wanted to or not but there was still a feeling of I needed to be very, very certain in myself before I got referred to the gender clinic because that’s a long waiting list and I didn’t wanna make anyone else wait for something I wasn’t very sure of”* (110). Other young people discussed shifting from a more concrete conceptualisation of gender identity linked to gendered behaviours, to a more abstract conceptualisation of gender as a societal construct, with autism helping them to understand gender more clearly. One young person described this shift in conceptualising gender “*I didn’t have the whole typical girl personality, even though now I don’t believe that your gender really influences your personality that much*” and said because of their autism they had a good understanding of gender *“…it’s not very complicated to me so I’m able to distinguish sex and gender and it’s not like I look at it and get confused*” (101).

Some parents highlighted their children’s differences in social understanding when making a gender transition. Parents described their children’s difficulties in making sense of social challenges that come with being gender diverse. For example, one parent said *“I do wonder looking back – beauty of hindsight – how much the autism impacted on his ability to understand his gender, A. in relation to himself and B. in relation to everybody else.” (216).* Parents described their children’s high expectations of others’ abilities to adjust and use the correct pronouns immediately after coming out, one parent elaborated; “*I think it’s other people that annoy him more and more like being frustrated by the ignorance…It’s got to be really hard for [child] not to stand up for himself as well at the moment cause it’s still quite early days…”*(110). This quote demonstrates the how the young person’s difficulties in understanding others’ perspectives and navigating social relationships increased his frustration when others treated him as the wrong gender.

## Discussion

This study is the first to explore the lived experience of gender dysphoria from the perspective of autistic young people and their parents. We captured key similarities and differences in the way that autistic young people and their parents conceptualised the young person’s distress and needs. Namely, young people described experiencing unpleasant feelings which they understood as being about gender incongruence and looked to alleviate these feelings through a gender transition, which parents were sometimes unsure about. Moreover, young people and their parents often focused on different needs; while young people were focused on meeting their gender-related needs, parents focused on meeting autism-related needs. Our findings resonate with the existing literature about the lived experience of gender dysphoria in non-autistic [17] and autistic young people [32].

Our study provides insight into the autistic experience of gender dysphoria, particularly when comparing that experience of autistic young people to the general population. A recent meta-synthesis of 12 qualitative studies investigated the lived experience of gender dysphoria in young people and adults aged between 12 and 29 years [17]. The meta-synthesis captured the distress and disconnection that young people felt in their bodies, as described by the autistic young people in this study. However, in our study, young people placed significant emphasis on their need for a physical transition, which was present but less emphasised within the meta-synthesis. Autistic participants also described distress due to the interaction of sensory sensitivities and gender incongruence, for example when experiencing periods, which was not described by the general population included in the meta-synthesis. Moreover, Jessen et al. [17] identified a theme around sexuality, which we did not find in our research. These differences may be due to the older age of participants included in the meta-synthesis compared to our study, or differences in autistic people. Jessen et al. [17] described how young people with gender dysphoria experienced vague feelings of difference alongside a need to explore their gender diversity, and that over time, their gender identity became coherent. Autistic young people described similar experiences, which are captured within our theme *the centrality of different identities and needs*, with the added layer for autistic young people of needing to make sense of the social differences connected to autism. An important new finding from our study is that while autistic young people were making sense of their differences through the lens of gender, similarly to the young people in the meta-synthesis, parents were more often considering their child’s differences through the lens of autism. Thinking styles associated with autism were considered to both ‘help’ and ‘hinder’ understanding of gender identity; some young people felt that being autistic helped them to understand the concept of gender, while some participants highlighted how black and white thinking could make it difficult to understand their gender identity and others’ responses to this. It should be noted that autism is a heterogenous condition, and just as thinking styles vary significantly across autistic individuals, autism thinking styles and gender cognition will interact (or not) in numerous ways. Participants in our study hoped for their gender identities to be affirmed by others and could become frustrated when this did not happen quickly enough, and Jessen et al. [17] similarly described how transgender young people experienced difficulties when their gender identity was misrecognised.

Our findings align with those of Strang et al. [32], indicating many similarities in the experience of gender dysphoria in young autistic people in the USA and the UK. The key differences came from our inclusion of parents in the study, and the points of difference between views of young people and parents. One such difference was which identity was prioritised. Young people were often more focused on their gender identity rather than autism, and did not tend to talk about other facets of their identities, although they were not asked to so during the interview. This lack of focus on autism is concordant with previous qualitative findings that young autistic people sometimes choose to distance themselves from their autism diagnosis [22]. Macleod et al. [22] found that autistic students sometimes prioritised their identities as students over their autism identities, which may be considered a less socially accepted identity. Furthermore, there is evidence that autistic young people can attempt to hide, or camouflage, their differences linked to autism [39]. Considering these previous findings, it is perhaps not surprising that autistic young people were reluctant to think about autism in the interviews. Moreover, young people expressed concerns that being autistic might cause other people to question their gender identities, which could have increased any desires to distance themselves from their autism diagnosis. Parents, however, often felt that autism was a salient and important part of their child’s gender experience, and highlighted how feelings of difference linked to autism might have compounded experiences of gender dysphoria. Given the mismatch between parent–child views on the role of autism, and the potential impact of feelings of difference on distress, it may be useful to provide post-diagnostic support groups to autistic young people which help them make sense of their autism diagnoses. Such groups could encourage a balanced perspective on their autism [14], and help them to make sense of negative social experiences such as bullying [16], while tackling shame and stigma linked to being autistic, as well as separately accessing gender-focused support for those experiencing gender dysphoria.

Young people and parents discussed their needs as autistic individuals in gender clinic settings, and often described difficulties in communicating with their gender clinicians and mental health clinicians. This fits with findings in the adult literature that communication between neurotypical and autistic people is less effective than communication between autistic people [10], given that most clinicians are not autistic themselves. Such communication issues are likely compounded by the difficulties participants identified in describing and communicating about their emotional experiences associated with gender dysphoria. Participants also described difficulties with practicalities and the physical environment at clinics which could have been adapted to meet their needs, for example by having a quiet waiting area, consistent clinician, local clinics, and sensory considerations in terms of lighting, noise, and temperature in clinics. The barriers and potential adaptations we identified in this study align with the barriers to autistic people accessing mental health settings found in previous research [1], and as described by autistic adults with gender dysphoria [9]. Moreover, these adaptations fit with recently published research indicating that higher rates of executive function difficulties in autistic and gender diverse young people may contribute to difficulties in accessing gender care [34, 35], and so will require adaptations in health settings. Gender clinics and mental health settings would, therefore, benefit from making autism adaptations to both clinician communication styles and the physical environment.

Parents and young people sometimes agreed about their aims to help alleviate gender dysphoria, with some parents stepping into an advocate role to help their child access a gender clinic, with the hope of gaining access to hormone treatments. Some parents were concerned about their children making irreversible decisions about their bodies and emphasised the need for their child’s gender identity to be certain and stable; young people also emphasised the need for certainty in their gender identities. These findings demonstrate the importance of the family context when young people are seeking support for gender dysphoria, in line with previous findings in gender diverse young people, where family support is associated with better psychological well-being [28, 41]. Parents tended to focus on their child’s needs linked to autism. This may have been because from the parent perspective, the challenges which were seen as being associated with autism were more urgent as compared to the challenges associated with gender dysphoria. For example, in the case of parenting a child who was expressing distress through challenging behaviours, navigating these situations would likely require significant time and effort from the parent as well as the young person [24], and the parent might be more likely to link such behaviours to autism rather than gender. It was noteworthy that despite the political and polarised debates surrounding transgender health for young people in the UK [6], parents and young people alike were willing to discuss the complexities of being autistic and transgender. Although young people focused more on gender identity than autism, both young people and their parents were willing to consider the relationship between their autism and gender identities, as well as how the experience of gender dysphoria linked to mental health needs. Participant accounts were not as polarised or stereotyped as one might believe from engaging with the online and media debates.

In this study, we recruited a higher proportion of sex-assigned females than sex-assigned males; this is in contrast to the higher rates of autism in sex-assigned males than females [21]. However, this pattern is in line with higher numbers of sex-assigned females attending gender clinics [19]. In the UK Gender Identity Development Service (GIDS), 69% of adolescents aged 13–17 were assigned female at birth according to the referral data for 2020–2021, when recruitment for this study was ongoing [12]. Moreover, the most recently available data on rates of autism diagnosis at GIDS, from 2015, indicate equivalent rates of autism diagnosis, with autism diagnoses in 15.4% of those assigned female at birth, compared to 14.6% of those assigned male at birth [25]. Therefore, the higher proportion of sex-assigned females compared to males recruited in this study is reflective of the current gender clinic context. Nonetheless, it is a limitation of this study that we recruited a slightly higher proportion of sex-assigned females than are seen in GIDS, and that we were not able to interview more participants who were assigned male at birth. At present, there is no clear evidence explaining the sex-ratio in gender clinic referrals, let alone that of autistic young people. Future studies should explore the perspectives of this trans-masculine and trans-feminine participants separately to better understand the specific experiences of each group.

To conclude, we investigated the lived experience of gender dysphoria in autistic young people, from their perspective and that of their parents. The experience of gender dysphoria in autistic young people shared some similarities with the experience of those without autism, including significant distress which was understood over time as being related to gender incongruence, and frustration when the young person’s gender was not affirmed by others. Features which were specific to autistic young people were the interaction of autism features and gender dysphoria, including sensory sensitivities and different cognitive styles. Furthermore, there were added complexities of navigating the young person’s needs linked to autism, and divergences in parent and young person viewpoints as to the salience of autism and gender identity. Future research should use longitudinal methods to investigate the development of diverse gender identities in autistic young people, outcomes of social and physical gender transition in this population, and interventions supporting a positive sense of autism identity in autistic young people more broadly.

## Data Availability

Consent was not gained to share the full transcripts outside of the research team.
